# Single‐stage autograft revision for failed ligament advanced reinforcement system (LARS) anterior cruciate ligament reconstruction: Improved clinical outcomes at a minimum 5‐year follow‐up

**DOI:** 10.1002/jeo2.70381

**Published:** 2025-08-05

**Authors:** Vasileios S. Akrivos, Antonios Koutalos, Ioannis Nastas, Nifon Gkekas, Pavlos Akritidis, Evangelos Gatos, Michael Hantes

**Affiliations:** ^1^ Department of Orthopaedic Surgery and Musculoskeletal Trauma, Faculty of Medicine, School of Health Sciences University of Thessalia Larissa Greece

**Keywords:** anterior cruciate ligament reconstruction (ACLR), ACL revision, ligament advanced reinforcement system (LARS), synthetic grafts

## Abstract

**Purpose:**

To evaluate mid‐term clinical outcomes and intraoperative findings in patients undergoing single‐stage revision after failed ligament advanced reinforcement system (LARS) anterior cruciate ligament reconstruction (ACLR).

**Methods:**

This retrospective study evaluated patients who underwent ACL revision surgery following initial reconstruction using the LARS device. Clinical assessments included the Tegner activity scale, Lysholm Knee score, and International Knee Documentation Committee (IKDC) scores, recorded preoperatively and at a minimum follow‐up of 5 years. Preoperative imaging was conducted to assess tunnel widening, alignment, and the presence of arthritic changes. Intraoperative evaluations included arthroscopic inspection of the synovium, menisci, and cartilage. Synovial biopsies were obtained for histological analysis of inflammation.

**Results:**

Twenty‐five patients were included in the study. Clinical scores demonstrated significant improvement in Tegner activity scale (*p* = 0.0006), Lysholm Knee score (*p* = 0.0001) and IKDC score (*p* = 0.0001) following revision surgery, with a mean follow‐up duration of 7.8 years (SD = 2.13). Preoperative imaging revealed early arthritic changes in 52% of patients. Intraoperative findings showed that all patients exhibited synovial membrane inflammation, with a 100% incidence of synovitis. Additionally, 68% of patients presented with Stage III or IV chondral lesions according to the ICRS classification.

**Conclusions:**

Single‐stage revision ACLR using autografts led to significant clinical improvement after LARS ACLR failure, with a mean follow‐up of 7.8 years. All cases during revision demonstrated synovial inflammation, with a high prevalence of chondral lesions and early arthritis. While these findings may point to a potential association between synthetic grafts and degenerative joint pathology, causality cannot be established, as degenerative changes are known to occur following failed ACL reconstructions regardless of graft type.

**Level of Evidence:**

Level IV.

**Abbreviations**
ACL: anterior cruciate ligament
ACLR: anterior cruciate ligament reconstruction
BPTB: bone–patellar tendon–bone
CRP: C‐reactive protein
ESR: erythrocyte sedimentation rate
ICRS: International Cartilage Repair Society
IKDC: International Knee Documentation Committee
IRB: Institutional Review Board
LARS: ligament advanced reinforcement system
LET: lateral extra‐articular tenodesis
MCL: medial collateral ligament
PET: polyethylene terephthalate
PROMs: Patient Reported Outcome Measures (PROMs)
ROM: range of motion
SD: standard deviation
WBC: white blood cell (count)

## INTRODUCTION

Anterior cruciate ligament (ACL) injuries represent one of the most common knee injuries with an estimated incidence of 200,000 per year in the United States [8, 10, 21]. These injuries can result in knee instability, and functional limitations of the knee joint, making anterior cruciate ligament reconstruction (ACLR) surgery a crucial intervention for restoring young and active patients' quality of life and athletic activity [17, 19]. Over the years, advancements in surgical techniques and graft options have expanded the choices available to both surgeons and patients. Autologous tendons harvested from the patient represent the graft of choice and the gold standard for the majority of the surgeons [14, 26]. Allograft tendons represent an option in primary ACLR for older patients and in revision ACL surgery [6]. In the past, another alternative, the synthetic grafts have emerged as a compelling option due to their potential to address some of the limitations associated with autografts [12]. Synthetic grafts, made from artificial materials, provide benefits such as immediate availability, reduced donor site morbidity, and shorter surgical times. However, concerns have been raised about their safety and long‐term outcomes, with reported failure rates ranging from 5% to 33%, which are higher than those associated with hamstring, bone–patellar tendon–bone (BPTB), and quadriceps grafts [18, 29]. Moreover, studies have reported that synthetic ligaments are associated with chronic synovitis, high complication rates of 66%, re‐rupture rates of 27.8%, and a high incidence of degenerative osteoarthritis at long‐term follow‐up [7, 9, 11, 16, 27, 28, 29, 30, 31]. Additionally, concerns have been raised about the use of synthetic grafts for ACLR, particularly due to unfavourable clinical outcomes following revision surgeries [30]. These issues have prompted to questions about the long‐term impact of synthetic grafts on the knee joint.

The aim of this study was to evaluate the mid‐term clinical results in patients who underwent revision surgery following ACLR using the synthetic ligament advanced reinforcement system (LARS) device in primary reconstruction. A secondary aim was to assess knee inflammation, cartilage lesions and Kellgren ‐Lawrence arthritic changes during the revision stage.

We hypothesised that revision ACL surgery using patient autografts would improve clinical outcomes following failed primary reconstruction with the LARS device. Additionally, we anticipated that during the revision procedure, patients would demonstrate synovial inflammation, cartilage lesions, and arthritic changes within the affected knee joint.

## METHODS

This retrospective study was conducted on patients who underwent revision ACLR between 2012 and 2018, with a minimum follow‐up period of five years. Inclusion criteria were: patients who had previously undergone primary ACLR using a isolated LARS device; patients presenting with one or more of the following persistent symptoms—knee instability, pain, recurrent effusions, or oedema—that had not responded to nonoperative treatment; and patients who underwent revision ACLR with autografts at our institution. Exclusion criteria were: patients who had undergone additional surgical procedures on the affected knee after the index LARS ACLR but prior to revision; patients with a history of inflammatory arthritis (e.g., rheumatoid arthritis); patients who had received intra‐articular knee injections, in order to avoid potential confounding effects on synovitis; and patients with active knee infection at the time of revision surgery. All participants provided written informed consent prior to their inclusion in the study. IRB approval was obtained (Approval Number: 2955).

All patients included in the study had undergone primary ACLR using LARS device between 2008 and 2013. Comprehensive clinical assessments were conducted, including examinations such as the anterior drawer test at 90° of flexion, anterior translation measured by the KT‐1000 under maximal manual force by the same examiner, and the Lachman test performed at 20° of flexion. Both the Lachman test and Anterior Drawer sign were categorised as either negative or positive in this study. Patients were also administered the Tegner activity scale to assess their activity level, as well as the Lysholm Knee score and International Knee Documentation Committee (IKDC) score to evaluate patient reported outcome measures (PROMs). The scores were assessed pre‐operatively and at 5 years minimum postoperatively. Any postoperative complications were also systematically recorded during follow‐up visits. Furthermore, imaging investigations, including X‐rays, were utilised pre‐operatively to evaluate the stage of arthritis based on Kellgren–Lawrence staging and to ensure a thorough evaluation of each case. Knee alignment is routinely assessed preoperatively in all ACL revision cases. Accordingly, full‐length lower limb radiographs were obtained for all patients who had undergone primary LARS device implantation. Joint inflammation was assessed arthroscopically, based on the presence of hyperaemic, thickened, and hypertrophic synovial membrane. Synovial biopsies were obtained during the procedure, placed in formaldehyde solution, and sent to the histology department for standard independent processing and staining. Additionally, the menisci were evaluated and treated based on the type of tear and tissue quality. The cartilage was assessed using the International Cartilage Repair Society (ICRS) classification.

### Surgical technique

All revision ACL reconstructions were performed arthroscopically by one senior surgeon. All of the patients underwent a one‐stage revision procedure. In all cases ipsilateral hamstrings or patellar tendon autograft was used for ACLR. Graft selection was based on tunnel anatomy, patient age, and intraoperative surgeon judgement; when the femoral tunnel was closer to the native ACL footprint, a BPTB graft was preferred to optimise fixation and provide additional stability through bone‐to‐bone healing, particularly in younger patients. Femoral fixation was performed using a suspensory device, while tibial fixation was performed with both an interference screw and an additional post fixation bicortical screw. In the case of BPTB reconstruction, the medial third of the patellar tendon was harvested. This harvested portion, along with the attached bone blocks, was then fixed in the femur and tibia using an interference screw.

**Figure 1:**
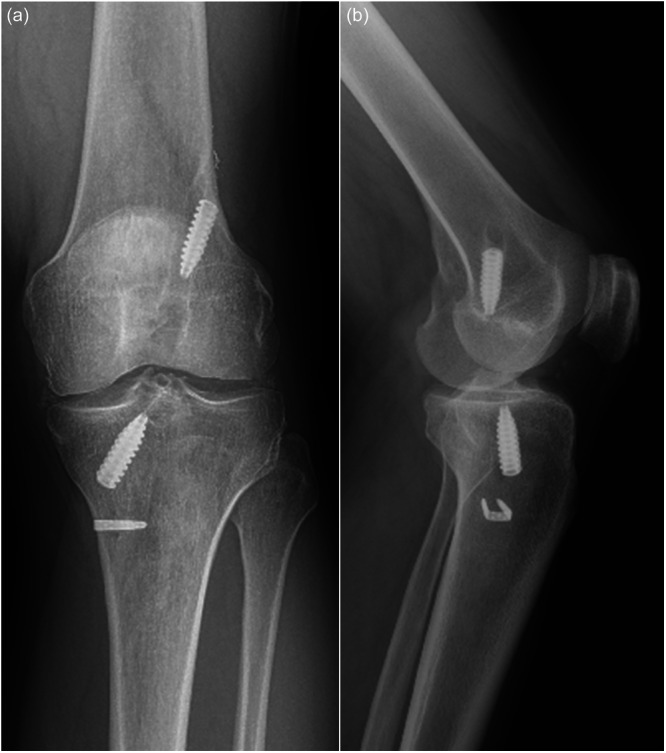
An anteroposterior (a) standing weight‐bearing radiograph and a lateral (b) radiograph of the left knee, obtained 6 years after ligament advanced reinforcement system (LARS) device implantation, revealed Grade III arthritic changes.

Typically, in the femoral part, the interference screw from the previous surgery was not removed since it was placed in a non‐anatomical position using an outside‐in technique (Figure 1). In all cases, the previously tunnel was malpositioned and therefore did not interfere with the new one. An inside‐out technique was used to create the new femoral tunnel, targeting the native ACL footprint with a guide positioned 7 mm offset from the posterior condyle. As for the tibial part removal of the interference screw was performed always. A new tibial tunnel (starting from a more medial position) was created if the previous one was malpositioned. Otherwise, the old tunnel was used. An outside‐in technique was used to create the new tibial tunnel, with a tibial guide set to a 55‐degree tunnel trajectory.

A modified Lemaire lateral extra‐articular tenodesis (LET) was performed in all patients who tested positive on the pivot shift test. Additionally, three more patients, who presented with knee recurvatum, underwent lateral tenodesis despite not exhibiting a positive pivot shift test.

An extensive synovectomy of the anterior compartment, was undertaken in all cases while synovectomy of the posterior compartment (through posterolateral and posteromedial portals) was performed when synovitis was present in the posterior compartment.

Meniscal repair was performed using various techniques, including all‐inside, inside‐out, or a combination of both. Microfracture was performed in case of Grade III and IV cartilage lesions. In all of our patients a drain was placed for one day.

### Rehabilitation

Postoperative rehabilitation following revision ACLR with autografts prioritised pain management and swelling reduction, with gradual weight‐bearing using crutches and range of motion exercises focusing on knee extension. By 3 weeks, patients progress to full weight‐bearing, closed kinetic chain exercises, and patellar mobilisations. From 6–12 weeks, emphasis shifts to full knee flexion, balance training, and advanced strengthening. At 3‐6 months, sport‐specific drills, agility training, and running are introduced. By 6–12 months, functional testing assesses readiness for return to sport, focusing on maintaining strength, flexibility, and injury prevention. Regular follow‐ups ensure optimal outcomes. If meniscal repair is performed, knee flexion is limited to 90° for the first 6 weeks, accompanied by partial weight‐bearing. For chondroplasty, partial weight‐bearing is implemented for 8 weeks.

### Statistical analysis

Paired *t*‐tests were employed to compare preoperative and postoperative scores on the Tegner activity scale, Lysholm Knee score and IKDC scores. Statistical significance was set at *p* < 0.01. The Tegner activity scale was reported as median and inter‐quartile range (Q1–Q3). Data for other continuous variables were presented as means with standard deviations, and categorical variables as frequencies and percentages.

## RESULTS

Our study involved 25 patients, whose characteristics are presented in Table 1. Mean follow‐up time was 7.8 years (SD = 2.1, range 5–12 years). PROMs demonstrated significant improvement following revision surgery (Table 2).

**Table 1:** Patient characteristics.

Characteristics	Number of patients (%)
Mean age [range in years] (SD)	38 [20–51] (7.75)
Male/female	15 (60)/10 (40)
Right/left	21 (84)/4 (16)
Instability	21 (84)
Pain	18 (72)
Effusion	20 (80)
Mean time from initial surgery to revision in years [range in months] (SD)	4.2 [24–84] (1.25)
Pivot shift positive	18 (72)
Meniscal tear	17 (68)
Medial/lateral meniscus	11 (44)/8 (32)
Partial meniscectomy	7 (28)
Meniscal repair	10 (40)
Chondral lesions (Grade III–IV)	18 (72)
Kellgren–Lawrence Stage I–II	13 (52)
Kellgren–Lawrence Stage III	2 (8)
Revision with hamstring	16 (64)
Revision with BPTB	9 (36)
Lateral tenodesis	21 (84)
Anterior synovectomy	25 (100)
Posterior synovectomy	14 (56)
Loose body removal‐ posterior compartment	2 (8)

Abbreviations: BPTB, bone–patellar tendon–bone; SD, standard deviation.

**Table 2:** Pre‐operatively and post‐operatively PROMs.

	Pre‐operatively mean (SD)[95% confidence interval	Post‐operatively mean (SD)[95% confidence interval]	*p*‐Value
Median Tegner activity score (Inter‐quartile range Q1–Q3)	2 (1.5–3)	3 (2–3)	*p* = 0.0006
Lysholm score	62 (6.9) [59.2–64.7]	95 (8.7) [91.5–98.4]	*p* = 0.0001
IKDC 2000	63.2 (8.7) [59.7–66.6]	91.2 (7.5) [88.2–94.1]	*p* = 0.0001

Abbreviations: IKDC, International Knee Documentation Committee; PROMs, patient reported outcome measures.

During revision surgery, no patients exhibited clinical signs of knee infection, and all preoperative inflammatory markers—including C‐reactive protein (CRP), erythrocyte sedimentation rate (ESR) and white blood cell count (WBC)—were within normal limits.

All of our patients had been operated elsewhere for the initial surgery. None of the patients exhibited significant malalignment necessitating osteotomy.

Intraoperatively removal of the artificial ligament, was successfully performed in all patients. Failure was confirmed intraoperatively by the presence of a ruptured LARS device in all cases. The removal of the synthetic graft remnants revealed the lack of macroscopic integration of the ligament at the bone‐ligament interface. Moreover, tunnel malposition was identified in all patients, predominantly at the femoral site. The observed malposition of the tunnel necessitated the creation of a new tunnel.

The meniscal tears were categorised as complex, horizontal, or vertical longitudinal types. The location and grading of the chondral lesions are summarised in Table 3. In all cases the area of cartilage lesions was less than 2 cm^2^. Microfractures were performed in all 18 patients.

**Table 3:** Location and grading of chondral lesions (ICRS classification).

Location	Grade 3 lesions	Grade 4 lesions
Lateral femoral condyle	6	1
Medial femoral condyle	8	2
Combined (one patient)	1 (lateral) + 1 (medial)	—

Abbreviation: ICRS, International Cartilage Repair Society.

**Figure 2:**
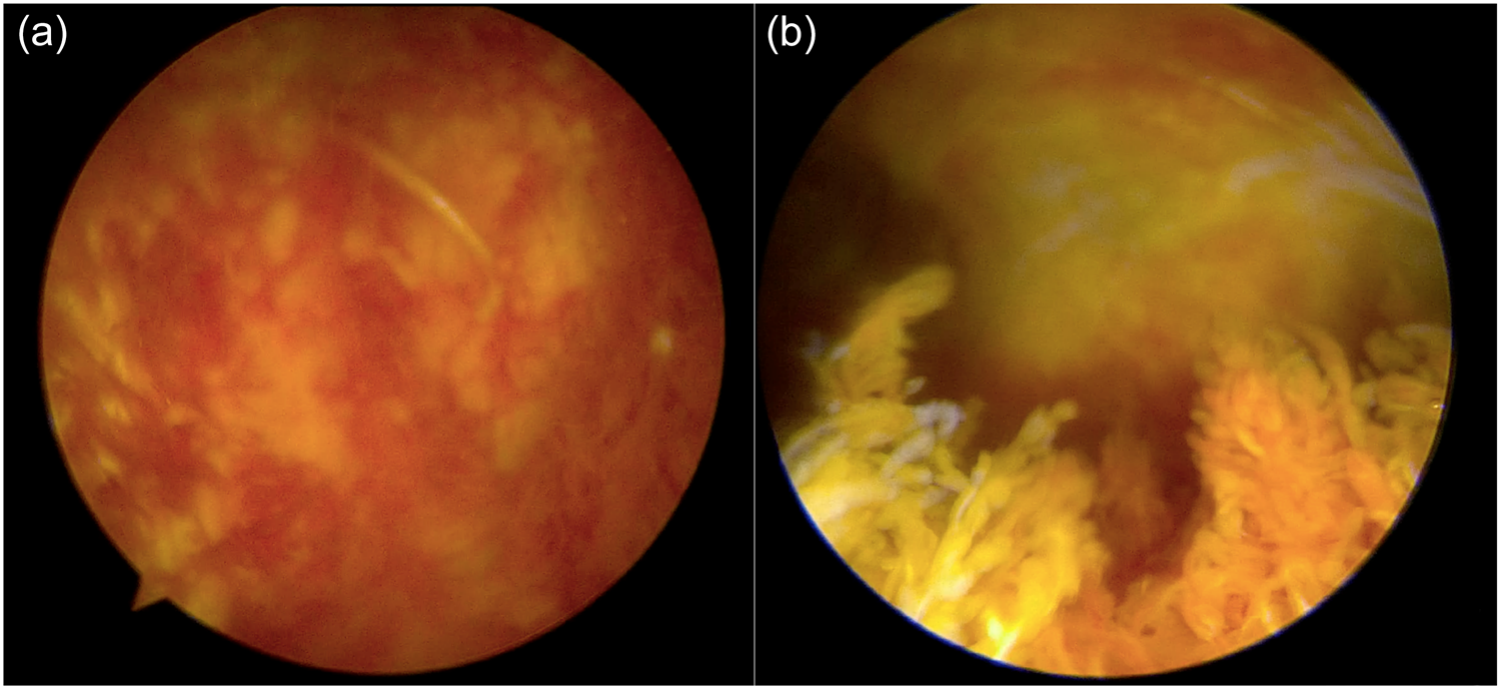
The patients are positioned supine, with the viewing perspective from the anterolateral portal on the right side. (a) Inflammated synovium in the medial wall of the suprapatellar pouch. (b) Inflammated synovium in the suprapatellar pouch and the presence of inflammated villi.

The two patients with Stage III changes had the longest intervals between primary and revision surgery (6 and 7 years) (Figure 1).Histological examination of the synovial tissue affirmed the arthroscopic findings of synovitis. The main findings were hyperplasia and hypertrophy of the synovial tissue, exhibiting a characteristic cellularity indicative of chronic inflammation, marked by the prevalence of multinucleated giant cells typical of a foreign body reaction (Figure 2a,b).

Only one patient (4%) required reoperation during the follow‐up period. The procedure was performed under local anaesthesia to remove the tibial post‐fixation screw, which was causing localised discomfort due to its subcutaneous prominence. The patient had low subcutaneous fat, and the screw was irritating during direct contact with external objects. The removal resolved the issue without impacting graft function or clinical outcomes.

Two patients (8%) developed postoperative range of motion (ROM) deficits, with limitations in both extension and flexion. Specifically, both patients exhibited an extension loss of 10° and a flexion deficit of 20° at final follow‐up. Notably, both individuals had Kellgren–Lawrence grade III degenerative changes at the time of revision surgery, which likely contributed to joint stiffness despite adherence to the rehabilitation protocol. No other complications such as infection, deep vein thrombosis, neurovascular injury, or arthrofibrosis were observed in the remaining cohort.

One patient (4%) experienced graft failure, which occurred following a traumatic fall from a bike approximately 4.5 years after the revision surgery. Clinical evaluation and imaging confirmed rupture of the reconstructed ACL graft, making this the only case of failure in the series. This results in a graft failure rate of 4% over a mean follow‐up of 7.8 years.

## DISCUSSION

Our study presented significantly improved clinical outcomes at a mean follow‐up of 7.8 years in patients treated with autografts after failed ACLR using the LARS device. Additionally, it revealed a striking 100% presence of inflammation in the synovial membrane in all 25 patients who underwent ACLR using synthetic grafts. Furthermore, 72% of the patients exhibited Stage III or IV chondral lesions according to the ICRS classification system, 52% showed signs of early arthritis and 8% of advanced arthritis, thereby confirming our hypothesis. While it is not possible to determine from this study whether the observed chondral lesions and arthritic changes were primarily related to the LARS device, tunnel malposition, or pre‐existing joint pathology, their presence even at a relatively short mean time to revision (4.2 years) is concerning.

During the 1980s, synthetic ligaments gained popularity as an alternative to biological grafts due to advantages such as reduced donor site morbidity, immediate strength, and quicker rehabilitation. Materials used included carbon fibre, polypropylene, Dacron, and polyester, either as standalone grafts or to augment biological reconstructions. However, early clinical experience revealed significant complications, including synovitis, foreign body reactions, chronic effusions and long‐term joint degeneration [2, 3, 5, 11, 20, 23, 24, 25]. Rushton et al. [24] reported knee inflammation following carbon fibre implants, while Wilson et al. [33] noted lymphadenopathy after Gore‐Tex use. Several studies using Dacron grafts reported synovitis, sterile effusions, and particle debris within the joint [11, 20, 32]. Klein and Jensen [15] described advanced osteoarthritic changes four years after Dacron implantation, characterising artificial ligaments as a model of iatrogenic joint degeneration. Ventura et al. [30], in a 19‐year follow‐up of athletes with PET ligaments, found a high rate of degenerative osteoarthritis, raising concerns about the long‐term safety of synthetic ACL grafts.

The LARS device is a synthetic ligament made of polyethylene terephthalate (PET) fibres, designed to provide early mechanical stability while supporting tissue ingrowth. It features two distinct segments: an intraosseous portion with longitudinal fibres bound by a transverse knit, and an intra‐articular portion composed of parallel longitudinal fibres twisted at 90° [29]. Although the LARS technique allows for anatomical tunnel placement, early recommendations favoured isometric positioning and stump preservation, which may have influenced tunnel placement practices in earlier LARS surgeries.

Di Benedetto et al. [7] reported that histological examination of synovial tissue in failed LARS reconstructions revealed chronic synovitis with multinucleated giant cells. Polarised light microscopy identified birefringent LARS particles within the tissue, and wear debris was observed inside the cytoplasm of these cells. Analysis of the removed grafts showed a typical foreign body response and limited fibrovascular integration. The authors concluded that failure likely results from a combination of biological factors, such as poor tissue incorporation, and mechanical factors, including graft material properties and tunnel placement.

Similar findings were reported by Blakeney et al. [4], who analysed histology of LARS grafts retrieved after failure and found a severe foreign body reaction in 86% of cases (18/22), and a mild reaction in the remaining 14% (4/22). Tissue ingrowth was minimal in most ACL grafts, with only moderate ingrowth observed in a few cases. Likewise, Ambrosio et al. [1] described a case of severe foreign body reaction with extensive osteolysis of both femoral and tibial tunnels following ACLR with a LARS graft. Histological analysis revealed chronic inflammation, fibrosis, and foreign body giant cells with synthetic fibre inclusions.

Tulloch et al. [29] reported a high graft failure rate of 33.3% at a mean follow‐up of 7.8 years in patients who underwent ACLR using the LARS device. Although patient‐reported outcomes were favourable in cases where the graft remained intact, the overall failure rate led the authors to recommend against using the LARS device for primary ACLR. Tiefenboeck et al. [28] observed good patient‐reported outcomes at a 10‐year follow‐up, with 27.8% of patients suffering rerupture and 22.2% experiencing re‐rupture, also suggesting that the LARS device is not suitable for ACLR. Similarly, Smolle et al. [27] presented results at a mean follow‐up of 12.8 years, showing that while the LARS device provided good functional and quality of life outcomes, it also had a complication rate of 66%, including graft failures and reactive synovitis. They emphasised that the use of this device should be approached with great caution.

Contrary to our results, Ventura et al. [31] reported on 14 patients with an average follow‐up of 4.2 years after revision surgery for failed ACLR using synthetic ligaments. They found that the improvement in clinical scores was not statistically significant, suggesting that revision surgery did not improve clinical outcomes or influence the natural history of the condition. Niki et al. [22] presented a study on 20 patients with a 2.8‐year follow‐up who underwent revision ACLR after synthetic ligament failure, using BPTB grafts. They found that the ACLR revision yielded favourable results in terms of IKDC grade, Lysholm score, and anteroposterior stability.

The clinical implications of this study are significant. Firstly, it highlights the role of the revision surgery following LARS ACLR. Although the Tegner score remained low, the primary goal of revision surgery was not return to high‐demand sport, but to address instability, recurrent effusions, and pain. The significant improvements in Lysholm and IKDC scores reflect successful symptom relief and functional recovery, despite underlying joint pathology. Secondly, it underscores serious concerns regarding the use of synthetic grafts for ACLR. All patients in the study demonstrated marked synovial inflammation, with a high incidence of chondral lesions and early arthritic changes. Although causality cannot be definitively established, these findings suggest an association between synthetic graft use and intra‐articular inflammation, cartilage damage, and joint degeneration. Synthetic grafts may not provide joint stability and biological integration required for successful ACLR, particularly in younger or high‐demand patients. Therefore, they are not recommended as a first‐line option for intra‐articular ligament reconstruction. However, their use may still be appropriate in select cases involving extra‐articular structures, such as the medial collateral ligament (MCL) [13].

## LIMITATIONS

This study has several limitations. Its retrospective design, small sample size and no priori power analysis reduce both statistical power and the generalisability of the findings. The lack of a control group, such as patients undergoing revision ACL reconstruction after failure of biological grafts, limits the ability to directly compare outcomes and interpret the specific impact of synthetic graft failure on joint pathology. Additionally, the exact time interval between clinical failure of the LARS graft and revision surgery was not routinely documented and could not be analysed, which may represent a potential confounding factor in the interpretation of outcomes. Assessment of cartilage damage and synovial inflammation relied on intraoperative observations and histological analysis, without the support of advanced imaging techniques such as MRI. Detailed data regarding histological processing, staining techniques, and formal scoring criteria were not available, and the histological findings are based on descriptive pathology reports. Although all patients exhibited synovial inflammation and degenerative changes at the time of revision, the relationship with prior LARS device use remains associative and does not establish causation. Future prospective randomised studies with larger cohorts, standardised histological protocols are needed to better define the long‐term effects of synthetic grafts.

## CONCLUSION

Single‐stage revision ACLR using autografts led to significant clinical improvement after LARS ACLR failure, with a mean follow‐up of 7.8 years. All cases during revision demonstrated synovial inflammation, with a high prevalence of chondral lesions and early arthritis. While these findings may point to a potential association between synthetic grafts and degenerative joint pathology, causality cannot be established, as degenerative changes are known to occur following failed ACL reconstructions regardless of graft type.

## AUTHOR CONTRIBUTIONS

Vasileios S. Akrivos: Contributed to study conception and design, data collection, data analysis, and drafting of the manuscript. Antonios Koutalos: Assisted in study design and revised the manuscript. Ioannis Nastas: Participated in data collection, surgical assistance, and patient follow‐up evaluations. Nifon Gkekas: Performed statistical analysis and contributed to manuscript editing. Pavlos Akritidis: Participated in data collection, literature review, and manuscript formatting. Evangelos Gatos: Performed follow‐up evaluations and contributed to manuscript preparation. Michael Hantes: Principal investigator and main surgeon; led study conception and design, performed all surgical procedures, supervised the research process, and approved the final manuscript. All authors read and approved the final version of the manuscript and agree to be accountable for all aspects of the work.

## CONFLICT OF INTEREST STATEMENT

The authors declare no conflict of interest.

## ETHICS STATEMENT

This study was approved by the Scientific Board of the University Hospital of Larisa (Approval Number: 2955), and all patients provided informed consent prior to participation, in accordance with the Declaration of Helsinki.

## Data Availability

The data that support the findings of this study are available from the corresponding author upon reasonable request.
